# Substrate specificity characterization for eight putative nudix hydrolases. Evaluation of criteria for substrate identification within the Nudix family

**DOI:** 10.1002/prot.25163

**Published:** 2016-10-01

**Authors:** Vi N. Nguyen, Annsea Park, Anting Xu, John R. Srouji, Steven E. Brenner, Jack F. Kirsch

**Affiliations:** ^1^ Molecular and Cell Biology DepartmentUniversity of CaliforniaBerkeleyCalifornia94720; ^2^ Graduate Program in Comparative BiochemistryUniversity of CaliforniaBerkeleyCalifornia94720; ^3^ Plant and Microbial Biology DepartmentUniversity of CaliforniaBerkeleyCalifornia94720; ^4^ Present address: Molecular and Cellular Biology DepartmentHarvard UniversityCambridgeMA02138

**Keywords:** kinetics, physiological substrate, Nudix, substrate screening

## Abstract

The nearly 50,000 known Nudix proteins have a diverse array of functions, of which the most extensively studied is the catalyzed hydrolysis of aberrant nucleotide triphosphates. The functions of 171 Nudix proteins have been characterized to some degree, although physiological relevance of the assayed activities has not always been conclusively demonstrated. We investigated substrate specificity for eight structurally characterized Nudix proteins, whose functions were unknown. These proteins were screened for hydrolase activity against a 74‐compound library of known Nudix enzyme substrates. We found substrates for four enzymes with *k*_cat_/*K*_m_ values >10,000 M^−1 ^s^−1^: Q92EH0_LISIN of *Listeria innocua* serovar 6a against ADP‐ribose, Q5LBB1_BACFN of *Bacillus fragilis* against 5‐Me‐CTP, and Q0TTC5_CLOP1 and Q0TS82_CLOP1 of *Clostridium perfringens* against 8‐oxo‐dATP and 3'‐dGTP, respectively. To ascertain whether these identified substrates were physiologically relevant, we surveyed all reported Nudix hydrolytic activities against NTPs. Twenty‐two Nudix enzymes are reported to have activity against canonical NTPs. With a single exception, we find that the reported *k*_cat_/*K*_m_ values exhibited against these canonical substrates are well under 10^5^ M^−1 ^s^−1^. By contrast, several Nudix enzymes show much larger *k*_cat_/*K*_m_ values (in the range of 10^5^ to >10^7^ M^−1 ^s^−1^) against noncanonical NTPs. We therefore conclude that hydrolytic activities exhibited by these enzymes against canonical NTPs are not likely their physiological function, but rather the result of unavoidable collateral damage occasioned by the enzymes' inability to distinguish completely between similar substrate structures. Proteins 2016; 84:1810–1822. © 2016 The Authors Proteins: Structure, Function, and Bioinformatics Published by Wiley Periodicals, Inc.

**Abbreviations**
PPi: pyrophosphate
PPase: inorganic pyrophosphatase
APase: alkaline phosphatase
MDCC: N‐(2‐(1‐maleimidyl)ethyl)−7‐(diethylamino)coumarin‐3‐carboxamide
PBP: phosphate binding protein
Pi‐sensor: PBP labeled with MDCC

## INTRODUCTION

The Nudix protein superfamily is vast and diverse.^1^, ^2^ It comprises about 50,000 members in the Nudix clan (Pfam ID: CL0261) of the Pfam database (version 27.0),^3^, ^4^ and all members share a characteristic ∼130 amino acid beta‐grasp domain architecture^5^ classified as the Nudix fold (SCOPe v2.03 SUNID 55810, SCCSID d.113;^6^, ^7^). Many Nudix proteins are pyrophosphohydrolases. They catalyze the hydrolysis of **nu**cleoside **di**phosphates linked to some other moiety, **X**.^1^ These Nudix hydrolases are typically characterized by a sequence of 23 amino acids **[Gx_5_Ex_7_REUxEExGU]**, where **U** can be Ile, Leu, or Val, and **x** represents any amino acid. The common structural theme amongst Nudix hydrolases is that the active site contains a magnesium binding site that serves to recognize the pyrophosphate linkage common to all Nudix substrates.^4^ In the Pfam database, non‐hydrolase proteins are also classified under the Nudix clan. For these proteins, one or a few conserved residues of the 23‐amino‐acid Nudix box vary. They do still share a characteristic ∼130 amino acid beta‐grasp domain architecture^5^ classified as the Nudix fold (SCOPe v2.03 SUNID 55810, SCCSID d.113^6^, ^7^).

Although Nudix proteins were originally proposed to catalyze reactions that “sanitize” the nucleotide pool, and thus act as “housecleaning” enzymes,^1^ they are now known to exhibit a wide range of activities apparently delimited only by the common recognition of a pyrophosphate bond of the substrate. As of July 2013, 171 Nudix proteins had been experimentally characterized (J. R. Srouji *et al*. Submitted), which serve a variety of cellular functions, including messenger RNA decapping,^8^ alternative mRNA polyadenylation,^9^ 3′→5′ RNA exonuclease activity,^10^ isopentenyl pyrophosphate isomerization,^11^ ADP‐ribose responding calcium channel gating,^12^ ADP‐ribose responding transcriptional regulation,^13^ SIRT1 or related deacetylase regulation,^14^, ^15^ and hydrolysis of a large group of nucleoside diphosphate derivatives.^16^ Specifically, a large subset of enzymes in this family hydrolyzes potentially mutagenic NTPs, such as 8‐oxo‐dGTP,^17^ 2‐OH‐dATP,^18^ and 5‐methyl‐UTP.^19^

Although sequences for about 50,000 Nudix family genes are available, X‐ray or NMR structures of only 78 Nudix proteins are found in the PDB database (Feb 1st, 2013).^20^ Some experimental characterization data are available for ∼2/3 of these 78 proteins, but the identities of the true physiological substrates for many of them are uncertain. The eight proteins selected for functional characterization in this study were chosen because: (1) X‐ray structures were available; (2) none had been characterized experimentally; and (3) they fall into well separated clades of the Nudix family tree, based on sequence and structure analysis (J. R. Srouji *et al*. Submitted). Thus substrate identification for these enzymes would be expected to provide a resource that would enhance computational functional annotation of additional Nudix genes.

A screening library of 74 chemicals was assembled and initially 63 of these were divided into 11 groups that were screened as mixtures, and 11 chemicals were screened individually. Chemicals from the most active groups were selected and screened individually. The most reactive compounds were assayed carefully to determine the kinetic parameters. The medium throughput assays were carried out with a phosphate sensor fluorescence assay.^21^

Finally, many of the previously characterized enzymes, as well as some of those investigated here, were found to exhibit catalytic activity on a variety of substrates with disparate values of kinetic constants. Thus, identifying the true physiological substrates can often be a challenge. In the Discussion Section, we suggest a series of criteria to facilitate this process.

## MATERIALS AND METHODS

### Materials

One functionally characterized Nudix protein in this study, B9WTJ0_STRSU, was encoded in a plasmid constructed by the Joint Center for Structural Genomics. The other seven were encoded in plasmids supplied by the New York SGX Research Center for Structural Genomics. All eight were purchased from the PSI:Biology‐Materials Repository using the Clone IDs provided in Table 1.

**Table 1:** Nudix Hydrolases Functionally Characterized in This Article

UniProt Entry Name	UniProt AC	Clone ID	Species	PDB ID
A0ZZM4_BIFAA	A0ZZM4	BbCD00291849	*Bifidobacterium adolescentis*	3FJY^62^
Q5LBB1_BACFN	Q5LBB1	BfCD00292626	*Bacteroides fragilis*	3GWY^63^
Q9K704_BACHD	Q9K704	BhCD00312276	*Bacillus halodurans*	3FK9^64^
Q0TTC5_CLOP1	Q0TTC5	CpCD00291844	*Clostridium perfringens*	3FCM^65^
Q0TS82_CLOP1	Q0TS82	CpCD00291806	*Clostridium perfringens*	3F6A^66^
Q92EH0_LISIN	Q92EH0	LiCD00291913	*Listeria innocua*	3I9X^67^
Q8PYE2_METMA	Q8PYE2	MmCD00291907	*Methanosarcina mazei*	3GRN^68^
B9WTJ0_STRSU	B9WTJ0	SsCD00104454	*Streptococcus suis*	3O8S^69^

The PBP A197C^22^ *E. coli* expression strain was a gift from Dr. Martin Webb (National Institute for Medical Research, UK). The BL21 (DE3) *Escherichia coli* expression strain was purchased from Invitrogen (Carlsbad, CA).

The Novagen BugBuster protein extraction reagents were from EMD4Biosciences (Gibbstown, NJ, USA). The Gene Jet Plasmid Miniprep Kit was from Fermentas (Glen Burnie, MA). Amicon Ultra‐15 and −4 NMWL 10,000 and 0.22 μm PES syringe membranes were from Millipore (Bedford, MA).

Modified nucleotides were purchased from TriLink Biotechnologies (San Diego, CA), Jena Bioscience (Jena, Germany), USB (Santa Clara, CA, USA), Fisher Scientific (Pittsburg, PA), and MP Biomedicals (Santa Ana, CA). MDCC, common biochemicals and enzymes were from Sigma‐Aldrich (St. Louis, MO).

Fast Protein Liquid Chromatography (FPLC) was performed on a BioLogic DuoFlow 10 workstation (from Bio‐Rad, Hercules, CA). The HisTrap HP liquid chromatography column was supplied by GE Healthcare (Piscataway, NJ). P_i_‐sensor assays were performed on either a FluoroMax‐4 spectrofluorometer (from HORIBA Jobin Yvon, Edison, NJ) or a GENios microplate reader (from Tecan, Switzerland).

### Nudix protein purification

The eight enzymes investigated here were purified with a His‐Tag protein purification protocol. The vector harboring Q8PYE2_METMA fused with the C‐terminal 6‐His tag was extracted from the storage strain of the PSI:Biology‐Materials Repository (kanamycin resistant, grown in LB medium) using the standard protocol of the Gene Jet Plasmid Miniprep Kit. The plasmid was transformed into BL21(DE3) cells.

Q8PYE2_METMA was expressed and purified as described by Harris *et al*.^23^ with the following changes: The BL21(DE3) cells bearing the pSGX3‐Q8PYE2_METMA plasmid were grown overnight in LB at 37°C, diluted with LB (1:50) to 4 L, and grown at 37°C for about 2.5 h until Abs_600_ was between 0.5 and 1.0. IPTG was added to a final concentration of 0.5 m*M* to induce protein production for 2 h. The cells were harvested by centrifugation at 4500*g*, at 4°C for 20 min. The cell pellets were stored at −80°C. Frozen cell pellets corresponding to 0.5 L of cell culture were lysed with BugBuster reagents as described in the kit protocol. Cell debris was removed by centrifugation at 5000*g*, at 4°C for 20 min.

The cell lysate was filtered through a 0.22 μm PES syringe membrane, and the filtrate applied to a HisTrap HP column equilibrated with 50 m*M* Tris‐HCl buffer, pH 7.6 containing 10 m*M* imidazole. Q8PYE2_METMA was eluted with 0–100% gradient of buffer containing 500 m*M* NaCl and 500 m*M* imidazole. Q8PYE2_METMA fractions were combined and concentrated to < 500 μL by Amicon filtration. The final preparation of Q8PYE2_METMA was > 95% pure as judged from SDS‐PAGE. These fractions were combined, concentrated to ∼ 100 μ*M*, divided into 20‐μL aliquots, and stored at −80°C.

A0ZZM4_BIFAA, Q9K704_BACHD, B9WTJ0_STRSU, Q0TTC5_CLOP1, Q0TS82_CLOP1, Q92EH0_LISIN and Q5LBB1_BACFN were similarly purified. Additionally, 1 m*M* DTT was included throughout the purification procedure for Q5LBB1_BACFN, Q92EH0_LISIN, B9WTJ0_STRSU, Q0TTC5_CLOP1, Q0TS82_CLOP1, Q9K704_BACHD and A0ZZM4_BIFAA to protect their cysteine residues.

### Preparation of the p_i_‐sensor

Fluorescently labeled phosphate binding protein (PBP) was expressed and purified as described in Xu *et al*.^21^

### Mass spectrometric analysis of selected substrates

Nanospray mass spectrometry was performed on 16 of the 74 substrates for which high activities were found in the initial screening assay. They were ADP‐ribose, ADP‐glucose, cADP‐ribose, 5‐Me‐dCTP, 5‐MeOH‐dCTP, 5‐OH‐dCTP, 5‐Me CTP, CTP, dCTP, 8‐oxo‐dATP, 8‐oxo‐dGTP, 8‐oxo‐GTP, dGTP, GTP, 3'‐dGTP, and ddGTP. Negative mode nano spray was used for 5‐Me‐dCTP, 5‐MeOH‐dCTP, 5‐OH‐dCTP, 8‐oxo‐dATP, 8‐oxo‐dGTP, 8‐oxo‐GTP, dGTP, GTP, 3'‐dGTP, and ddGTP. Positive mode nano spray was used for ADP‐ribose, ADP‐glucose, cADP‐ribose, CTP, and dCTP. All substrates were >90% pure.

### Enzyme assays

The P_i_‐sensor kinetic assays were performed on a GENios microplate reader (reaction volume = 100 μL) for initial screening and with a FluoroMax‐4 spectrofluorometer (reaction volume = 500 μL) for accurate determination of the kinetic parameters. The standard reaction mixture contained: 10 m*M* Tris‐HCl, pH 7.6, 1 m*M* MgCl_2_, 5–10 μ*M* PBP‐MDCC (depending on the concentration of background phosphate introduced by the substrates impurities), 0.05 U/mL of yeast pyrophosphatase (PPase) where pyrophosphate was one of the products, or 1 U/mL of alkaline phosphatase (APase), where a nucleoside monophosphate was a Nudix enzyme product. Experiments were done in all cases to verify that sufficient coupling enzyme was present to ensure that the rates of reaction were linearly dependent on the concentration of the Nudix hydrolase. Nudix enzymes concentrations ranged from 1–100 n*M*. The mixtures were incubated at 37°C and monitored continuously for 30 min on the microplate reader or for 5 min on the spectrofluorometer. (GENios: *λ*_ex_ 425 nm, *λ*_em_ 465 nm, gain 50, 100 cycles, 37°C; FluoroMax‐4: *λ*_ex_ 430 nm, slit width 1 − 2.5 nm, *λ*_em_ 465 nm, slit width 1–5 nm, 37°C).

**Figure 1:**
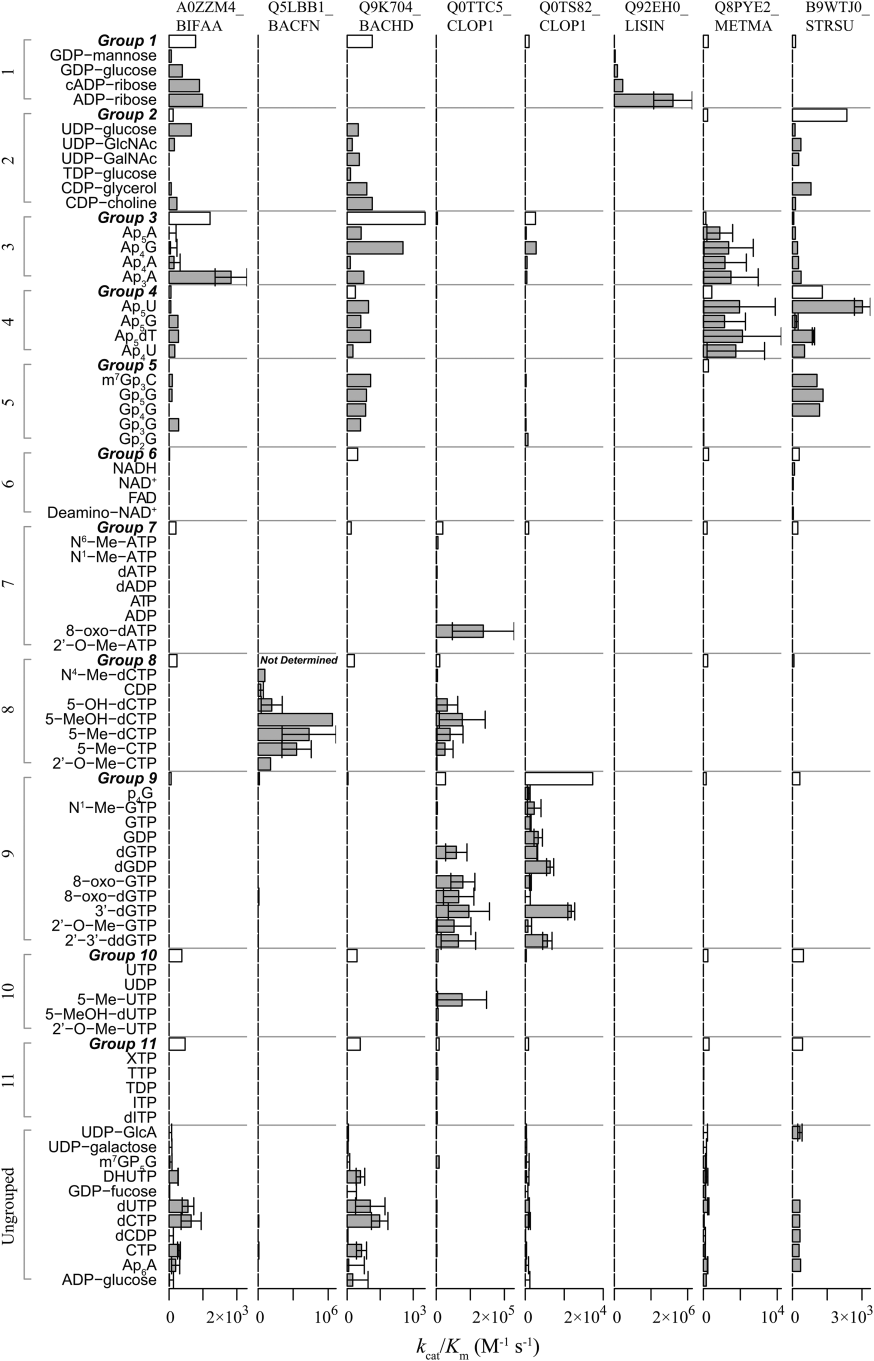
Substrate specificity screening of eight putative Nudix hydrolases against a 74‐compound library by P_i_‐sensor assay. Approximate *k*_cat_/*K*_m_ values (M^−1^ s^−1^) are reported with error bars. Each reaction was carried out at pH 7.6 and 37°C with each substrate at 5 μ*M*. Enzyme concentrations varied from 1 to 100 n*M*. Sixty‐three of the potential substrates were mixed into 11 groups and screened as indicated by the numbers in the left brackets. The eleven substrates shown in the “ungrouped” bracket were initially screened individually in one plate. The kinetic values for compounds that were assayed only in the specified group are reported with the grouped activity, which thus represents the upper limits for each component substrate (white bars). Substrates that were assayed individually are reported with mean (gray bars) and standard errors if tested more than three times. The X axes are scaled linearly and are different for each enzyme.

A screening library of 74 commercially available putative substrates was assembled primarily from compounds that had been shown to be active with one or more Nudix hydrolases (J. R. Srouji *et al*. Submitted). Chemicals were grouped initially by structural similarity and by considerations of the necessity and choice of either alkaline phosphatase or inorganic pyrophosphatase as a coupling enzyme in the P_i_‐sensor assay.^21^ Typically the 74 substrates were screened in about 22 wells of the microplate. Sixty three of the substrates were assembled into 11 groups; 11 others were assayed individually because of their high free phosphate background (Fig. 1, left brackets and numbers).

Each substrate concentration was 5 μ*M* in both grouped and individual screenings. The compounds from individual screening that showed significant activity over background (600 RFU above background) were assayed from 0 to 20 μ*M*, with enzyme concentrations varied from 1 to 100 n*M*. The kinetics were typically evaluated for each substrate concentration three or more times, with enzyme concentrations varying from 0.26 to 52 n*M*, to determine the values of the Michaelis‐Menten parameters.

### Data processing

The regression calculations below were performed with scripts written in R.^24^ *P_i_*‐sensor standard curves for normalizing fluorescence readings were obtained by titrating inorganic phosphate under the same conditions as were used in the experimental reactions. Fluorescence readings were plotted against reaction time, and the linear regions of the plots (RFU/s) yielded initial velocities, *v*_i_ (μ*M* P_i_/s). Plots of *v*_i_/[*E*_0_] were fit to the Michaelis‐Menten equation:

$$v_{i}=\frac{k_{cat}\;\left[S\right]}{K_{m}+\left[S\right]}$$

or its transformation:

$$\frac{v_{i}}{\left[E_{0}\right]}=\frac{\frac{k_{cat}}{K_{m}}\;\left[S\right]}{1+\frac{\left[S\right]}{K_{m}}}$$

respectively, by nonlinear regression to yield values of *k*_cat_, *K*_m_ and *k*_cat_/*K*_m_. For some reactions, nonlinear regression failed to converge, or the standard errors for *k*_cat_ and *K*_m_ were comparable to the fitted values themselves; in these cases, only the *k*_cat_/*K*_m_ ratio was obtained by linear regression with a fixed intercept of zero:

$$\frac{v_{i}}{\left[E_{0}\right]}=\frac{k_{cat}}{K_{m}}\;\left[S\right]$$

Two equivalents of inorganic phosphate are ultimately produced for those reactions that initially yield pyrophosphate in the presence of inorganic pyrophosphatase. The values of *v*_i_ were corrected for this factor. Data from multiple trials were fitted into the same equation to yield weighted average values of the kinetic parameters.

## RESULTS

### Functionally characterized nudix proteins

Enzymes Q0TTC5_CLOP1 and Q0TS82_CLOP1 are from *Clostridium perfringens* (strain ATCC 13124/NCTC 8237/Type A), a Gram‐positive, spore‐forming, obligate anaerobic bacterium. Bacterial alpha toxin produced by *C. perfringens* is responsible for histotoxic infections, such as gas gangrene. There are 13 putative Nudix proteins in *C. perfringens* strain ATCC 13124, as annotated by UniProt (release 2013_12),^25^ none of which had been functionally characterized previously. Nudix proteins have been shown to facilitate pathogenicity in the host^26^ as well as enhancing virulence of the pathogen.^27^

Enzyme Q92EH0_LISIN is from *Listeria innocua* (strain CLIP 11262), a Gram‐positive, non‐spore forming bacillus, which is a facultative anaerobe. *L. innocua* is ubiquitous because it can survive in extreme pH and temperature.^28^ It is important because it is very similar to the food‐borne pathogen *Listeria monocytogenes*, but is non‐pathogenic. In UniProt release 2013_12,^25^ none of the functions of the nine putative Nudix proteins in *L. innocua* (strain CLIP 11262) is reported as having been functionally characterized.

Enzyme Q5LBB1_BACFN is from *Bacteroides fragilis* (strain ATCC 25285/NCTC 9343). *Bacteroides* species is a Gram‐negative obligate gut anaerobe. *B. fragilis* is the most frequent isolate from clinical specimens, and is regarded as the most virulent *Bacteroides* species.^29^ Eight genes from *B. fragilis* strain ATCC 25285 are annotated as coding for putative Nudix proteins by UniProt release 2013_12,^2^ ^5^ There are no experimental functional characterization data for any of them.

Enzyme A0ZZM4_BIFAA is from *Bifidobacterium adolescentis*, a gram‐positive organism that is non‐motile and often observed in a Y‐shaped form. The bacteria colonize human and animal intestinal tracts.^30^

Enzyme B9WTJ0_STRSU is from *Streptococcus suis*, a Gram‐positive bacterium. It is a pathogen of pigs and is also a causative agent for zoonotic disease.^31^

Enzyme Q9K704_BACHD is from *Bacillus halodurans*, a rod‐shaped Gram‐positive, spore‐forming soil bacterium that can survive in alkaline environments.^32^, ^33^

Enzyme Q8PYE2_METMA is from *Methanosarcina mazei*, a methane‐producing archaeon. *M. mazei* is a freshwater organism that can adapt to grow at elevated salinities.^34^

### Initial substrate screening

Figure 1 shows the results from substrate screening of 74 compounds for eight potential Nudix hydrolases, in the presence of the appropriate secondary enzyme, namely PPase or APase. Approximate *k*_cat_/*K*_m_ values (M^−1^ s^−1^) are reported with error bars when applicable. The substrate screening results presented here emerged from a two‐step strategy. The substrates were initially grouped by structural similarity and screened in groups. Secondly, those substrates from the most reactive group(s) were separated and screened individually. The substrate concentrations for each compound—both in the grouped mixtures and in the individual screenings—were 5 μ*M*. Sixty‐three compounds were initially divided into 11 groups, and screened in the mixtures. The mean *k*_cat_/*K*_m_ values of each group is represented by a white bar. Compounds that passed the initial screening in groups were assayed individually. Those activities are shown in grey bars. Compounds were not assayed individually in cases where the grouped activities were low (for example, Q0TS82_CLOP1 and group 11). Eleven compounds could not be grouped because of their high phosphate background, and were screened individually. Those activities are also depicted by grey bars.

High values for the specificity constant (*k*_cat_/*K*_m_ > 10,000 M^–^ 1 s^−1^) were found for Q0TTC5_CLOP1, Q0TS82_CLOP1, Q92EH0_LISIN and Q5LBB1_BACFN with at least one substrate, and these reactions were characterized extensively (see below).

Q8PYE2_METMA exhibits moderate activity toward dinucleotide polyphosphates, where at least one of the bases is adenine (Fig. 1, Q8PYE2_METMA, groups 3 and 4), with *k*_cat_/*K*_m_ values of *ca*. 5,000 M^−1^ s^−1^; however, Q8PYE2_METMA shows no preference with respect to the structure of one of the two bases. This is consistent with our previous report,^21^ where Q8PYE2_METMA was shown to have moderate activities toward Ap_3_A, Ap_4_A, and Ap_5_A, but has none with 8‐oxo‐dGTP.

B9WTJ0_STRSU catalyzes the hydrolysis of a variety of dinucleotide polyphosphates such as Ap_5_U and Gp_5_G. The *k*_cat_/*K*_m_ value for Ap_5_U is ∼10‐fold greater than that found for the other tested substrates of this group; however, all of their *k*_cat_/*K*_m_ values are <3000 M^–^ 1 s^−1^.

No significant activity was found for A0ZZM4_BIFAA or Q9K704_BACHD against any substrate tested, as all *k*_cat_/*K*_m_ values are < 1000 M^−1^ s^−1^.

Theoretically, the apparent *k*_cat_/*K*_m_ value of grouped activity should be equal to the sum of the *k*_cat_/*K*_m_ values from individual screening of the same group. This is, however, generally not true for the data presented in Figure 1. Part of the inconsistency can be explained by the large errors that are inherent in compound library screening exercises. Specific examples include the reactions of Q0TTC5_CLOP1 with 8‐oxo‐dATP, and of Q8PYE2_METMA with Ap_5_U. Further the microplate reader used here has lower precision than does the cuvette spectrofluorometer. Finally, some compounds in a grouped mixture might act as nonhydrolyzable substrate analogs, thus they behave as competitive inhibitors for an otherwise active substrate.

### Michaelis‐menten parameters for the most active substrates

**Figure 2:**
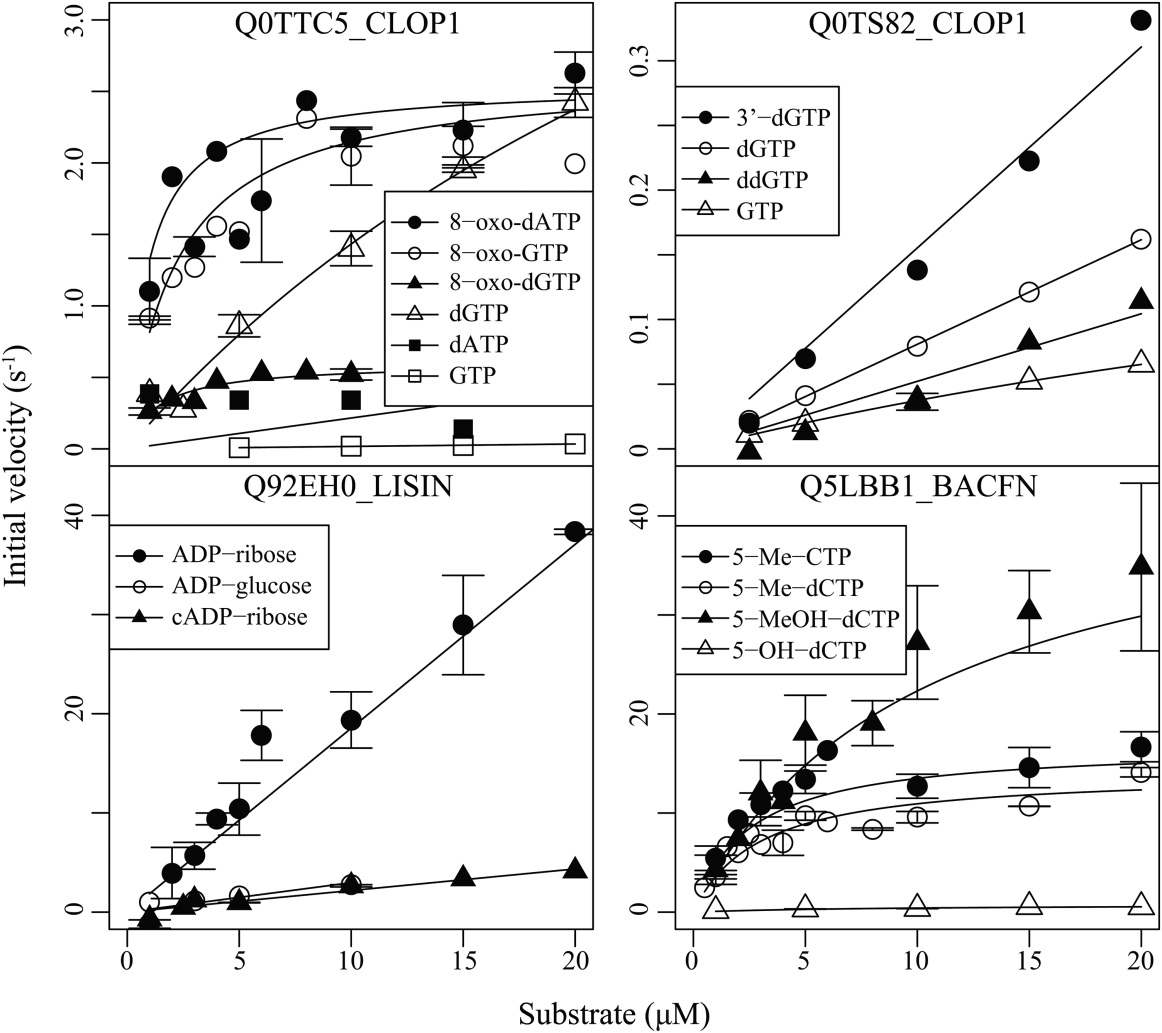
Kinetic characterization of 4 Nudix hydrolases against their most reactive substrates. The highly active substrates were identified in the initial plate reader screen. The reaction rates were monitored spectrofluorometrically in the *P_i_*‐sensor assay. The substrates specified in the legends are sorted by decreasing values of *k*_cat_/*K*_m_. Error bars are shown when where replicate determinations were carried out. All assays were performed at pH 7.6 and 37°C, with enzyme concentrations varying from 0.26 to 52 n*M*. The coupling enzymes employed are listed in Table II. The fitted curves were calculated by either linear or nonlinear regression, as specified in the methods section.

The enzyme‐substrate pairs with approximate *k*_cat_/*K*_m_ > 10,000 M^–^ 1 s^−1^ as identified from screening were further characterized to determine the kinetic parameters more precisely. Figure 2 shows the results for the reactions of Q0TTC5_CLOP1, Q0TS82_CLOP1, Q92EH0_LISIN, and Q5LBB1_BACFN with their most reactive substrates. The kinetic parameters and their standard errors are given in Table 2.

**Table 2:** Kinetic Parameters for Nudix Hydrolase Catalyzed Reactions

Enzyme Name^a^	Substrate	*k*_cat_ (s^−1^)	*K*_m_ (μ*M*)	*k*_cat_/*K*_m_ (M^−1^ s^−1^)
Q0TTC5_CLOP1	8‐oxo‐dATP	2.5 ± 0.1	0.93 ± 0.26	(2.8 ± 0.7) × 10^6^
	8‐oxo‐GTP	2.6 ± 0.1	2.2 ± 0.4	(1.2 ± 0.2) × 10^6^
	8‐oxo‐dGTP	0.60 ± 0.03	1.4 ± 0.3	(4.3 ± 0.9) × 10^5^
	dGTP	6.9 ± 1.5	38 ± 12	(1.8 ± 0.2) × 10^5^
	dATP	ND^b^	ND^b^	(2.2 ± 1.5) × 10^4^
	GTP	ND^b^	ND^b^	1,700 ± 50
Q0TS82_CLOP1	3′‐dGTP	ND^b^	ND^b^	(1.6 ± 0.1) × 10^4^
	dGTP	ND^b^	ND^b^	8,080 ± 40
	ddGTP	ND^b^	ND	5,200 ± 400
	GTP	0.26 ± 0.02	59 ± 6	4,400 ± 100
Q92EH0_LISIN	ADP‐ribose	ND^b^	ND^b^	(1.85 ± 0.08) × 10^6^
	ADP‐glucose	ND^b^	ND^b^	(2.97 ± 0.04) × 10^5^
	cADP‐ribose	ND^b^	ND^b^	(2.18 ± 0.05) × 10^5^
Q5LBB1_BACFN	5‐Me‐CTP	16 ± 3	2.5 ± 1.2	(6.7 ± 2.4) × 10^6^
	5‐Me‐dCTP	14 ± 1	3.0 ± 0.8	(4.8 ± 1.0) × 10^6^
	5‐MeOH‐dCTP	45 ± 11	10 ± 5	(4.4 ± 1.0) × 10^6^
	5‐OH‐dCTP	0.78 ± 0.11	9.2 ± 2.8	(8.5 ± 1.4) × 10^4^

a Reactions were carried out at pH 7.6 and 37°C. Inorganic pyrophosphatase was the coupling enzyme for Q0TTC5_CLOP1, Q0TS82_CLOP1 and Q5LBB1_BACFN, and alkaline phosphatase for Q92EH0_LISIN.

b ND, not determined as *k*_cat_/*K*_m_ was obtained from linear regression fitting.

#### Q0TTC5_CLOP1

The most reactive substrate tested for Q0TTC5_CLOP1 is 8‐oxo‐dATP with *k*_cat_/*K*_m_ = (2.8 ± 0.7) × 10^6^ M^−1^ s^−1^. This value approaches the diffusion‐controlled limit (see Discussion). The next most reactive substrates are in order: 8‐oxo‐GTP, which is about one‐third as reactive, followed by 8‐oxo‐dGTP, dGTP, dATP, and GTP. Based on considerations elaborated in the Discussion section, it is tentatively concluded that 8‐oxo‐dATP and 8‐oxo‐GTP are the target substrates for this housekeeping enzyme. Q0TTC5_CLOP1 discriminates variously between the ribose and deoxyribose moieties of the substrate; for example, the *k*_cat_/*K*_m_ ratio for 8‐oxo‐GTP >8‐oxo‐dGTP is 3, but it is 100 for dGTP versus GTP. Comparison of the *v*_i_/[E_0_] values in the absence of PPase (data not shown) indicated that Q0TTC5_CLOP1 cleaves the substrates mainly at the α‐β pyrophosphate bond.

#### Q0TS82_CLOP1

3′‐dGTP is the most reactive substrate for Q0TS82_CLOP1 with a *k*_cat_/*K*_m_ value = (1.6 ± 0.06) × 10^4^ M^−1^ s^−1^. This figure is not sufficiently large to warrant the conclusion that this compound is a physiological target for this enzyme. dGTP is half as reactive as 3'‐dGTP. ddGTP follows with about one‐third of the activity of 3'‐dGTP. The *v*_i_/[E_0_] values in the presence of PPase are substantially greater than those recorded in its absence (data not shown), indicating that Q0TS82_CLOP1 predominantly catalyzes the hydrolysis of the tested substrates at the α‐β pyrophosphate bond.

#### Q92EH0_LISIN

Q92EH0_LISIN is most reactive toward ADP‐ribose in the presence of APase with a *k*_cat_/*K*_m_ value of (1.85 ± 0.08) × 10^6^ M^−1^ s^−1^, ADP‐glucose and cADP‐ribose are 30% and about 13% as reactive, respectively.

#### Q5LBB1_BACFN

Q5LBB1_BACFN efficiently catalyzes the hydrolysis of 5‐substituted cytidine nucleotide triphosphates, that is, 5‐Me‐dCTP, 5‐MeOH‐dCTP, and 5‐OH‐dCTP (Fig. 2, Q5LBB1_BACFN). This enzyme does not discriminate between the 5‐Me (*k*_cat_/*K*_m_ = (4.8 ± 1.0) × 10^6^ M^−1^ s^−1^) and 5‐MeOH (*k*_cat_/*K*_m_ = (4.4 ± 1.0) × 10^6^ M^−1^ s^−1^) substitutions. However, its activity against 5‐OH‐dCTP is about 50‐fold lower (*k*_cat_/*K*_m_ = (8.5 ± 1.4) × 10^4^ M^−1^ s^−1^). Comparison of the *v*_i_/[*E*_0_] values in the absence of PPase indicated that Q5LBB1_BACFN cleaves the substrates mainly at the α‐β pyrophosphate bond.

Some of the kinetic parameter determinations presented have large standard errors, especially those for Q5LBB1_BACFN (36% for 5‐Me‐CTP). We performed control experiments to maximize the reproducibility of the assay, including diluting the enzyme stock with different protocols, repeating the experiment with different batches of enzymes, washing the cuvette extensively with nitric acid, stirring the reaction solution with magnetic bars, and so forth In total, we repeated the measurement of 5‐Me‐CTP activity on 6 different days and that of 5‐Me‐dCTP on 14 different days, respectively. However, large standard errors were found on each of the different days, and when using each of the different approaches above. Therefore, the large error bars (Fig. 2, Q5LBB1_BACFN)—representing the entirety of the experiments—were not due to any of the factors that were considered.

In summary, a total of eight putative Nudix hydrolases was screened for activity against our library of 74 demonstrated substrates for this group of enzymes. Three of the enzymes were found to exhibit *k*_cat_/*K*_m_ values of >10^6^ M^−1 ^s^−1^ for either ADP ribose or for a noncanonical NTP, and, by the criterion presented in the Discussion, can be reasonably assigned the designated physiological function. The highest activity for the fourth enzyme is hydrolysis of the noncanonical 3'‐dGTP, but the *k*_cat_/*K*_m_ value of 1.6 × 10^4^ M^−1 ^s^−1^ is too low to allow a confident assignment of this activity to this enzyme.

To explore whether protein structure might help the assignment of function for the eight newly assayed enzymes, we studied the structures of the proteins considered herein and their similarities to other structurally characterized Nudix proteins. The results were unrevealing.

## DISCUSSION

### Principles to identify the physiological substrates for Nudix enzymes

Historically, enzymes were usually identified by pursuing a predefined assay to the point of highest specific activity as the enzyme was purified in stages. The homogeneity of the purified protein was usually ascertained by the available technology, and a limited range of alternate substrates was sometimes investigated. It is now recognized that many enzymes have more than a single activity.^35^, ^36^

The results presented in this article report an exploration of possible substrates for eight putative Nudix hydrolases. However, the mere observation of significant catalytic activity with a given substrate does not necessarily serve to define it as the physiological target. Here we propose a set of criteria to help to achieve such target identification for Nudix enzymes. We argue that a diffusion‐controlled *k*_cat_/*K*_m_ value (ca. > 10^6^ M^−1^ s^−1^) is usually definitive. When *k*_cat_/*K*_m_ is much less than this figure, genetic evidence and, to a lesser extent, genomic methods (see below), may provide conclusive evidence for an assignment. Although many Nudix hydrolases are reported to have activities for canonical nucleoside triphosphates (Table 3), only one of these activities exhibits a *k*_cat_/*K*_m_ value > 10^5^ M^–^ 1 s^−1^, and almost all the others have values of <10^4^ M^−1 ^s^−1^ (Table 3). Furthermore, many enzymes capable of hydrolyzing canonical NTPs show higher activities to noncanonical NTPs with similar structures. We therefore conclude that the apparent activities against canonical NTPs likely represent collateral damage. This criterion could potentially be applied generally when assigning physiological functions to other proteins of this family. We used that gauge to assign probable physiological activities for the three putative Nudix hydrolases (Q0TTC5_CLOP1, Q92EH0_LISIN, and Q5LBB1_BACFN) functionally characterized in this article (Table 2).

**Table 3:** Likely Physiological Substrates for Nudix Proteins With Canonical NTP Hydrolase Activities

	Canonical NTP		Likely physiological substrate		
Uniprot Entry Name^a^	Substrate	*k*_cat_/*K*_m_ (10^−3^ × M^−1^ s^−1^)	Substrate	*k*_cat_/*K*_m_ (10^−3^ × M^−1^ s^−1^)	Non‐kinetic evidence
MUTT_ECOLI	dGTP	10^53^	8‐oxo‐dGTP	61,000^53^	Mutator strains show an increase in A:T‐C:G transversion^70^
Q9RRX6_DEIRA	GTP	90^71^	Ap_5_A	12,000^71^	
8ODP_HUMAN	dGTP	60^18^	2‐OH‐dATP	1,700^18^	
			8‐oxo‐dGTP		Complementation of E.coli MutT deficient cells^72^
NUDG_ECOLI	CTP	47^73^	5‐methyl‐dCTP	1,300^74^	
			2‐OH‐dATP		The gene knockout exhibited increased frequencies of spontaneous and H_2_O_2_‐induced mutations, including G:C‐T:A transversion, which is elicited by 2‐OH‐dATP; Over‐expression suppressed such mutations^75^
DIPP_ASFB7	GTP	1.0^76^	m7G‐mRNA		Hydrolysis of mRNA cap tethered to an RNA moiety (gel)^77^
NUDB_ECOLI	dATP	7.5^50^	DHNTP		Gene knockout reduced folate synthesis; restored by plasmid with the gene^51^
NUDT1_ARATH	TTP	15^78^	8‐oxo‐dGTP		Complementation of E. coli MutT deficient cells^78^
Q6MPX4_BDEBA	dGTP	23^79^	mRNA		Complementation of E. coli RppH deficient cells^80^
A0R2K6_MYCS2	dCTP	4.4^81^	8‐oxo‐dGTP		Complementation of E. coli MutT deficient cells^81^
NUDJ_ECOLI	GDP	5.4^82^	CF_3_‐, MeO‐HMP‐PP, MeO‐TPP^b^		Identified as one of the genes conferring resistance to bacimethrin or CF_3_‐HMP; Gene product hydrolyzed CF_3_‐HMP‐PP, MeO‐HMP‐PP, and MeO‐TPP, the previously identified toxic forms of the antibiotics Hydrolysis of HMP‐PP (genetic screening)^83^
YTKD_BACSU	dGTP	4.6^84^	mRNA		Gene knockout prolonged half‐life of plasmid‐encoded transcripts and reduced the yield of monophosphorylated RNA 5'ends; A wild‐type copy restored it to normal^85^
YJ9J_YEAST	GDP	4.4^52^	Oxy‐, oxo‐ThDP^c^		Gene knockout lowered oxythiamin resistance; over‐expression raised it^52^
NUD20_ARATH	GDP	0.54^52^	Oxy‐, oxo‐ThDP		Expression of the gene in S. cerevisiae YJ9J_YEAST deletant strain increased oxythiamin resistance^53^
TNR3_SCHPO	GDP	2^52^			
B4FMB8_MAIZE	GDP	0.33^52^			
Q7CX66_AGRT5	UTP	51^19^			
Q9HYD6_PSEAE	UTP	30^19^			
Q9A8K7_CAUCR	UTP	130^19^			
Y079_DEIRA	CDP	20^86^			
NUDI_ECOLI	dTTP	11^82^			
Q9RVP7_DEIRA	dGDP	1.7^87^			
MUTT2_MYCTU	dCTP	1.2^81^			

All of the Nudix enzymes that have had *k*_cat_/*K*_m_ values determined for at least one canonical NTP are included in this table. The probable physiological substrates for these were evaluated based on the criteria proposed herein and/or in the literature. The likely physiological substrate is unknown for several of the listed proteins.

a All of the listed enzymes have reported *k*_cat_/*K*_m_ values ≤ 1.3 × 10^5^ M^−1 ^s^−1^ for the most reactive canonical NTP investigated.

b 4‐amino‐2‐trifluoromethyl‐5‐hydroxymethylpyrimidine pyrophosphate, 4‐amino‐2‐methoxy‐ 5‐hydroxymethylpyrimidine pyrophosphate, 2'‐methoxythiamin pyrophosphate.

c Oxythiamin diphosphate, oxothiamin diphosphate.

A nearly diffusion‐controlled value of *k*_cat_/*K*_m_ may serve as a sufficient condition to identify a likely physiological substrate, because virtually every enzyme substrate encounter is catalytically productive, assuming that said enzyme has physical access to that substrate.^37^, ^38^ Enzymes that catalyze such reactions have been called “perfect” because they cannot be improved by further evolution.^39^ The observation of a significantly smaller *k*_cat_/*K*_m_ value means that the investigated compound may not be the physiological substrate for the enzyme, or that such low activity is acceptable for the enzyme's cellular role. In cases where the substrate is poorly hydrolyzed, the observed activity might provide some hint regarding the structure of the physiological substrate, which might be similar to that of the less active substrate.

It is also possible that the physiological substrate for a given Nudix hydrolase may exhibit a low value of *k*_cat_/*K*_m_, if for example, the enzyme were allosterically regulated. There are few reports of regulation of Nudix activity. The only biochemical evidence of allosteric regulation of Nudix enzymes is for the ADP‐ribose pyrophosphatase of *E. coli* (UniProt Entry Name: ADPP_ECOLI). Although this enzyme's *k*_cat_/*K*_m_ of 1.75 × 10^6^ M^−1^ s^−1^ for ADP‐ribose^40^ is not low, this parameter is increased by 8‐fold in the presence of glucose 1,6‐diphosphate.^41^

Functional assignments based on lower than diffusion‐controlled values of *k*_cat_/*K*_m_ may be ambiguous, due to either catalytic promiscuity of the enzyme or significant structural relationships among many substrates. The results of genetic probes (for example, gene knockouts, and complementation tests), alongside enzymatic assays, are often definitive, as they provide orthogonal information regarding the physiological role of the enzyme in the cellular environment. For example, prior to genetic analysis, the highest *k*_cat_/*K*_m_ value for any examined substrate reacting with *E. coli* RNA pyrophosphohydrolase (gene name: *rppH*; UniProt Entry Name: RPPH_ECOLI) was 2800 M^−1^ s^−1^ for Ap_5_A.^42^ The criteria introduced above would cast doubt on the assignment of this as the primary activity of this enzyme. Indeed, subsequent experiments showed that this enzyme cleaves the pyrophosphate entity from the 5′ triphosphate end of RNA to yield a pyrophosphate ion (note that this is different from mRNA decapping activity in eukaryotes, as the eukaryotic mRNA has a m^7^G cap at the 5′ end of RNA), and *in vivo* accelerates the degradation of transcripts^43^; these data support the contention that RNA is the physiological substrate of RppH.

In addition to experimental characterization, functional assignment of enzyme activity is currently facilitated by genomic methods including operon and protein family evolution analyses.^44^, ^45^, ^46^, ^47^ An illustrative example is *gmm* from *E. coli* (UniProt Entry: GMM_ECOLI), which has been designated as a GDP‐mannose mannosyl hydrolase.^48^ Both the low *k*_cat_/*K*_m_ value of 1600 M^−1^ s^−1^ for GDP‐mannose and biosynthetic considerations call the assignment into question. *gmm* is part of an operon responsible for the synthesis of colanic acid. The two genes immediately upstream and downstream of *gmm* encode a GDP‐fucose synthase (*fcl*) and a predicted colanic biosynthesis glycosyl transferase (*wcal*), respectively.^49^ Moreover, GDP‐mannose is synthesized by an enzyme coded by *cpsB* in the same operon, immediately downstream of *wcal*. It would be biologically wasteful for the same operon to also code for an enzyme that hydrolyzes GDP‐mannose, thus completing a futile cycle, although it cannot be ruled out as a regulatory mechanism.

### Activities observed for canonical NTPs may be the result of collateral damage

As of July 2013, 161 activities associated with 171 Nudix enzymes have been reported in a total of 192 papers (J. R. Srouji *et al*. Submitted). A total of 107 enzymes have Michaelis‐Menten kinetic parameters reported for at least one substrate. Twenty enzymes exhibit *k*_cat_/*K*_m_ values of 10^6^−10^7^ M^−1^ s^−1^ (37 enzyme‐substrate pairs); thus the physiological substrates of these proteins may be assigned with a high degree of confidence (see above). High quality genetic data support the assignments of another 51 of the 171 enzymes. Therefore, assignments are relatively secure for 71 of the 171 characterized Nudix enzymes.

Twenty‐two of the 171 have reported *k*_cat_/*K*_m_ values for the hydrolysis of canonical nucleotide triphosphates (NTPs), including (d)GTP, (d)ATP, (d)CTP, TTP, and UTP (Table 3). However, none of the *k*_cat_/*K*_m_ values is >10^5^ M^−1^ s^−1^, with the exception of one enzyme (Q9A8K7_CAUCR), which exhibits a *k*_cat_/*K*_m_ value of 1.3 × 10^5^ M^−1^ s^−1^ for UTP.^19^ It is uncertain whether UTP is or is not the physiological substrate. Notably, three of these 22 Nudix hydrolases do have *k*_cat_/*K*_m_ values > 10^6^ M^−1^ s^−1^ for noncanonical NTPs (MUTT_ECOLI, 8ODP_HUMAN, and NUDG_ECOLI), providing evidence that their true function may be to eliminate noncanonical NTPs, supporting Bessman's earlier conclusion.^1^ The activity against canonical NTPs is therefore likely collateral damage. Furthermore, functional assignments for 12 of these 22 enzymes have been secured from genetic experiments, as discussed below. Notably, not one of the physiological substrates for these 12 enzymes is a canonical NTP, despite the enzymes' having some activity against them. There is a good chance that the physiological substrates for the remaining putative Nudix hydrolases have yet to be discovered.

An illustrative example of an enzyme function secured with strong genetic evidence is *nudB* (UniProt Entry: NUDB_ECOLI), which was originally characterized to preferably hydrolyze dATP with *k*_cat_/*K*_m_ of 6,600 M^−1^ s^–^ 1.^50^ It was later found to hydrolyze DHNTP, the substrate of the committed step in folic acid synthesis, but the *k*_cat_/*K*_m_ value is not high enough (4.3 × 10^4^ M^−1^ s^−1^) for definitive function assignment based on the kinetic constant alone.^51^ However, the deletion of *nudB* led to a reduction in folate biosynthesis, which was completely restored by a plasmid carrying the same gene.^51^

Genetic evidence also establishes that YJ9J_YEAST and its homolog, NUD20_ARATH, catalyze the hydrolysis of oxo‐ and oxy‐thiamine diphosphate *in vivo*.^52^ Specifically, deletion of YJ9J_YEAST decreases the oxythiamin resistance of its host *Saccharomyces cerevisiae*, and overexpression of NUD20_ARATH restores the oxy‐thiamine resistance in YJ9J_YEAST‐deleted *S. cerevisiae*. However, the *k*_cat_/*K*_m_ values are only on the order of 1000 M^−1^ s^−1^ for oxo‐ and oxy‐thiamine diphosphate, while both enzymes show higher activity for the canonical substrate GDP.^52^ One explanation is that the low hydrolase activity for oxo‐ and oxy‐thiamine diphosphate suffices because thiamine diphosphate is not produced in the cell in high quantities. Therefore, neither high catalytic activity for the designated substrate nor the perfection of substrate selectivity is evolutionarily mandated.

It is likely that the activities observed for the hydrolysis of the canonical NTPs may simply be the result of unavoidable collateral damage. For example, the well‐characterized MutT from *E. coli* effects the hydrolysis of the potentially mutagenic, 8‐oxo‐dGTP with a *k*_cat_/*K*_m_ = 6.1 × 10^7^ M^−1^ s^−1^, but also catalyzes the hydrolysis of dGTP with a *k*_cat_/*K*_m_ = 1.0 × 10^4^ M^−1^ s^−1^.^53^ A factor of 10^3^ corresponds to 4.2 kcal/mol, which is about the degree of specificity that might be gained by a single optimally placed charged hydrogen bond.^54^ The crystal structure of the MutT complex with 8‐oxo‐dGMP shows that MutT strictly recognizes the overall conformation of the 8‐oxo‐guanine base through multiple hydrogen bonds.^55^ Greater distinctions between similar molecules by the hydrolase might be achievable, but evolutionary considerations argue that substrate optimization would progress only to the point where further differentiation provides little or no added biological advantage. DNA, RNA, and protein biosynthesis all require far more robust selection of the component monomers, but this is achieved subsequent to the committed steps by repair or editing mechanisms, respectively at the ultimate expense of further energy consumption.^56^

It does take considerable energy to make NTP and other naturally biosynthesized Nudix substrates, such as coenzyme A, NADH and FAD. Why would they be made only to be subsequently discarded in a futile cycle? While low values (<10^5^ M^–^ 1 s^−1^) of *k*_cat_/*K*_m_ observed with physiological substrates (for example, canonical NTPs, CoA and its derivatives, and NAD^+^) might indicate that the observed activity is not the major function of the enzyme, it has to be recognized that these molecules in addition to their major roles in central metabolism, have additional regulatory functions. For example, hydrolysis of NAD^+^ may be a means for regulating the size of the peroxisomal pool of nicotinamide coenzymes independently of those in other subcellular compartments in response to a change in available carbon source.^57^ Thus, their concentration levels must be carefully monitored and controlled.

While the data collected in Table 3 and elsewhere in this article support the contention that unregulated canonical NTP hydrolase activity is not a defining characteristic of any enzymatically characterized Nudix hydrolase, they do not allow the conclusion that it is never purposeful to drive the hydrolysis of canonical NTPs. For example, the enzyme, SAMHD1, which is induced by HIV infection is allosterically activated by dGTP, and converts dNTPs to the corresponding nucleosides and inorganic triphosphate, presumably to reduce the rate of viral replication.^58^ SAMHD1 is unrelated to the Nudix family.

### Newly functionally characterized nudix hydrolases

Prior to this investigation, 20 Nudix enzymes had been shown to have *k*_cat_/*K*_m_ values >10^6^ M^–^ 1 s^−1^ for at least one substrate. We screened eight new ones, whose structures are known, against our library, and found three (Q0TTC5_CLOP1, Q92EH0_LISIN, and Q5LBB1_BACFN) to have *k*_cat_/*K*_m_ >10^6^ M^−1^ s^−1^ for at least one substrate, expanding by 15% the group of characterized Nudix enzymes with high *k*_cat_/*K*_m_ values. These three Nudix enzymes are also the first reported characterized examples from their respective host organisms. Five of eight do not show significant activity against the compounds in the library; their true activity remains to be discovered.

Two of the previously functionally uncharacterized Nudix hydrolases, Q0TTC5_CLOP1 and Q5LBB1_BACFN, demonstrate higher *k*_cat_/*K*_m_ values toward mutagenic nucleoside triphosphates than for the closest canonical NTPs. Q0TTC5_CLOP1 distinguishes the mutagenic NTPs (for example, 8‐oxo‐dATP) from the canonical NTPs (for example, dGTP) with a 10‐fold difference in *k*_cat_/*K*_m_. Q5LBB1_BACFN is most active against a mutagenic nucleotide (5‐substituted (d)CTP), with *k*_cat_/*K*_m_ values are >10^6^ M^−1^ s^−1^. Q5LBB1_BACFN has much lower activity against the canonical NTPs, as was shown in the screening result (Fig. 1). The third enzyme, Q92EH0_LISIN, hydrolyzes ADP‐ribose, also with a *k*_cat_/*K*_m_ value >10^6^ M^–^ 1 s^−1^. Therefore, based on these nearly diffusion‐controlled specificity constants, it is likely that the physiological substrates for these three hydrolases are now identified.

Q0TS82_CLOP1 shows significant activity only for nucleoside triphosphates containing a guanine base (Fig. 1). Detailed kinetic analysis for four of these showed that the best substrate assayed is 3'‐dGTP (*k*_cat_/*K*_m_ ∼ 2 × 10^4^ M^–^ 1 s^−1^). There are no reports showing that this is a naturally occurring molecule, although it is possible that it may be formed following an aberrant reduction by ribonucleotide reductase.^59^, ^60^, ^61^

The approximate *k*_cat_/*K*_m_ values obtained for Q8PYE2_METMA, B9WTJ0_STRSU, A0ZZM4_BIFAA, and Q9K704_BACHD from the screening experiments are fairly low (<5,000 M^–^ 1 s^−1^); thus it is unlikely that the appropriate substrates have been identified.

Our characterization of these enzymes will aid the prediction of the functions of others in the Nudix protein family. However, the huge size of the superfamily and its functional plasticity mean that common approaches often yield misleading results and even the most sophisticated protein function prediction methods must be used with care and deftness in this superfamily.^88^

## Supporting information

Supporting Information
