# Supplementary material for: Substrate specificity characterization for eight putative nudix hydrolases. Evaluation of criteria for substrate identification within the Nudix family
Source: Proteins. 2016 Oct 1;84(12):1810–22. doi: 10.1002/prot.25163 (PMC5158307; doi:10.1002/prot.25163)
Supplement: Supplementary file 1 — Supporting Information [file PROT-84-1810-s001.pdf]

Supplementary Material
Table S1

Substrate abbreviations.

Abbreviation	Name
2'-3'-ddGTP	2', 3'-Dideoxyguanosine-5'-triphosphate
2-OH-dATP	2-hydroxy-deoxyadenosine-5'-triphosphate
2'-O-Me-ATP	2'-O-methyladenosine-5'-triphosphate
2'-O-Me-CTP	2'-O-methylcytidine-5'-triphosphate
2'-O-Me-GTP	2'-O-methylguanosine-5'-triphosphate
2'-O-Me-UTP	2'-O-methyluridine-5'-triphosphate
3'-dGTP	3'-Deoxyguanosine-5'-triphosphate
5-Me-CTP	5-methylcytidine-5'-triphosphate
5-Me-dCTP	5-methyl-2'deoxycytidine-5'-triphosphate
5-MeOH-dCTP	5-hydroxymethyl-2'-deoxycytidine-5'-triphosphate
5-MeOH-dUTP	5-Hydroxymethyl-2'-deoxyuridine-5'-triphosphate
5-Me-UTP	5-Methyluridine-5'-triphosphate
5-OH-dCTP	5-hydroxy-2'-deoxycytidine-5'-triphosphate
8-oxo-dATP	8-Oxo-2'-deoxyadenosine-5'-triphosphate
8-oxo-dGTP	8-Oxo-2'-deoxyguanosine-5'-triphosphate
8-oxo-GTP	8-Oxoguanosine-5'-triphosphate
ADP-glucose	Adenosine-5'-diphosphoglucose
ADP-ribose	Adenosine 5'-diphosphoribose
Ap_3A	P^1,P^3-Di(adenosine-5') triphosphate
Ap_4A	P1,P^4-Di(adenosine-5') tetraphosphate
Ap_4dT	P^1(-5'-Adenosyl)-P^4-[5'-(2'-deoxy-thymidyl)]-tetraphosphate
Ap_4G	P^1(-5'-Adenosyl)-P^4-(5'-guanosyl)-tetrataphosphate
Ap_4U	P^1(-5'-Adenosyl)-P^4-(5'-uridyl)-tetraphosphate
Ap_5A	P^1,P^5-Di(adenosine-5′) pentaphosphate
Ap_5dT	P^1(-5'-Adenosyl)-P^5-[5'-(2'-deoxy-thymidyl)]-pentaphosphate
Ap_5G	P^1(-5'-Adenosyl)-P^5-(5'-guanosyl)-pentaphosphate
Ap_5U	P^1(-5'-Adenosyl)-P^5-(5'-uridyl)-pentaphosphate
Ap_6A	P^1(-5'-Adenosyl)-P^6-(5'-adenosyl)-hexaphosphate
CDP-choline	Cytidine 5′-diphosphocholine
CDP-glycerol	Cytidine 5′-diphosphoglycerol
CF_3-HMP	4-amino-2-trifluoromethyl-5-hydroxymethylpyrimidine
CF_3-HMP-PP	4-amino-2-trifluoromethyl-5-hydroxymethylpyrimidine pyrophosphate
Deamino-NAD+	Nicotinamide hypoxanthine dinucleotide
dITP	2'-Deoxyinosine-5'-triphosphate
GDP-fucose	Guanosine 5′-diphospho-β-L-fucose
GDP-glucose	Guanosine 5'-diphosphoglucose
GDP-mannose	Guanosine 5'-diphospho-D-mannose
Gp_2G	P^1(-5'-guanosyl)-P^2-(5'-guanosyl)-diphosphate
Gp_3G	P^1(-5'-guanosyl)-P^3-(5'-guanosyl)-triphosphate
Gp_4G	P^1(-5'-guanosyl)-P^4-(5'-guanosyl)-tetraphosphate
Gp_5G	P^1,P^5-di(guanosine-5') pentaphosphate
DHNTP	7,8-Dihydroneopterin triphosphate
DHUTP	5,6-Dihydrouridine-5'-triphosphate
HMP-pp	4-amino-2-methyl-5-hydroxymethylpyrimidine pyrophosphate
ITP	Inosine-5'-triphosphate
MeO-HMP-PP	4-amino-2-methoxy- 5-hydroxymethylpyrimidine pyrophosphate
MeO-TPP	2'-methoxythiamin pyrophosphate
m^7Gp_3C	P^1-(5'-7-methyl-guanosyl)-P^3-(5'-cytidyl)-triphosphate
m^7GP_5G	P^1-(5'-7-methyl-guanosyl)-P^5-(5'-guanosyl)-pentaphosphate

N^1-Me-ATP	N^1-methyladenosine-5'-triphosphate
N^1-Me-GTP	N^1-methylguanosine-5'-triphosphate
N^4-Me-dCTP	N^4-Methyl-2'-deoxycytidine-5'-triphosphate
N^6-Me-ATP	N^6-methyladenosine-5'-triphosphate
oxidized-CoA	Coenzyme A, oxidized (CoA-S-S-CoA)
p_4G	Guanosine 5'-tetraphosphate
ppGpp	Guanosine-3',5'-Bisdiphosphate
PRPP	5-Phospho-D-ribose 1-diphosphate
TDP-glucose	Thymidine-5′-diphospho-α-D-glucose
UDP-acetylgalactosamine	Uridine 5′-diphospho-N-acetylgalactosamine
UDP-acetylglucosamine	Uridine 5′-diphospho-N-acetylglucosamine
UDP-galactose	Uridine 5'-diphosphogalactose
UDP-glucose	Uridine 5'-diphosphoglucose
UDP-glucuronic acid	Uridine 5'-diphosphoglucuronic acid
XTP	Xanthosine-5'-triphosphate
